# Western and Mediterranean Dietary Patterns and Physical Activity and Fitness among Spanish Older Adults

**DOI:** 10.3390/nu9070704

**Published:** 2017-07-06

**Authors:** Maria del Mar Bibiloni, Alicia Julibert, Emma Argelich, Raquel Aparicio-Ugarriza, Gonzalo Palacios, Antoni Pons, Marcela Gonzalez-Gross, Josep A. Tur

**Affiliations:** 1 Research Group on Community Nutrition and Oxidative Stress, University of the Balearic Islands, Guillem Colom Bldg, Campus, E-07122 Palma de Mallorca, Spain; mar.bibiloni@uib.es (M.d.M.B.); aliciajulibert@gmail.com (A.J.); eargelich15@gmail.com (E.A.); antonipons@uib.es (A.P.); 2 CIBEROBN (Physiopathology of Obesity and Nutrition CB12/03/30038), Instituto de Salud Carlos III, E-28029 Madrid, Spain; gonzalopalacios88@gmail.com (G.P.); marcela.gonzalez.gross@upm.es (M.G.-G.); 3 ImFINE Research Group, Department of Health and Human Performance, Faculty of Physical Activity and Sport Sciences (INEF), Technical University of Madrid, E-28040 Madrid, Spain; apariciougarriza.raquel@gmail.com

**Keywords:** physical activity, physical fitness, body composition, dietary pattern, older adults

## Abstract

**Objectives:** To assess prevailing food patterns, and its association with physical activity and fitness among Spanish older adults. **Methods:** Cross-sectional study in Spain, collecting data from a sample (*n* = 380; 54% female) aged 55–80 years (men) and 60–80 years (women) with no previously documented cardiovascular disease. Body weight, body fat and waist circumference were assessed. Physical activity performed was measured using the Minnesota Leisure-time Physical Activity Questionnaire (LTPA). Physical fitness was assessed using a validated physical fitness test battery. Food consumption was assessed by a validated semi-quantitative food-frequency questionnaire. Factor analysis identified two major dietary food patterns: “Western” (WDP) and “Mediterranean” (MDP) dietary patterns. **Results:** Participants in MDP’s fourth quartile were classified in the second (men) and third (men and women) tertile of LTPA. After adjusting for age, body fat, waist-to-height ratio, and METs, in both sexes, a negative significant association was found between 30-s Chair stand and 6-min walking test, a positive significant association was found between 30-m Gait speed and 8-foot Time Up-and-Go (except in men) tests with WDP. The 30-m Gait speed test was negatively associated with MDP in men. **Conclusions:** MDP is associated with more time spent on LTPA, and this association was independent of body composition and a fast gait speed in men. WDP is associated with slower gait speed and lower body strength, agility and aerobic endurance. MDP has protective effect on healthy physical fitness, and WDP may be a contributor to frailty.

## 1. Introduction

Between 1990 and 2010, global consumption of healthier foods and nutrients has modestly increased; however, consumption of unhealthy foods and nutrients has increased to a greater extent during the past two decades [1]. Emerging evidence suggests that low intake of certain micronutrients and protein could be a risk factor for frailty [2,3]. Several studies also found that adherence to the Mediterranean diet (MD) [4,5,6,7] or higher scores on the global dietary quality index [8,9] were associated with a lower risk of frailty or some of its components. Lately, significant positive associations between MD and percentage of free fat mass and leg explosive power has been demonstrated in healthy women, emphasizing the role of diet quality on the prevention of age-related loss of muscle [10].

The MD used to be sufficiently caloric and rich in vitamins and minerals, derived from vegetables and fruits, whole-meal cereals, nuts, virgin olive oil and fish, which made the risk of deficient micronutrient intakes quite infrequent [11]. Moreover, the total antioxidant capacity of the MD is probably higher than in other countries with a higher incidence of chronic diseases [12].

Adverse health outcomes including falls, hospitalization, institutionalization and mortality are consequence of impaired homeostatic reserve and reduced capacity to withstand stress, characterized by physical weakness, reduced physical activity and performance that usually go along with frailty [13,14,15]. Regular physical activity and fitness has been shown to improve health, and decrease risk of chronic diseases, frailty, disability, falls, and mortality [16,17]. Physical fitness seems to be similar to physical activity regarding to morbidity and mortality [18,19] but is more predictive of health outcomes than physical activity [17,18,20]. Moreover, physical activity and fitness are often used in the same sense in epidemiologic research; however, physical activity is assessed by means of self-reports, whereas physical fitness is usually more accurately measured [20].

An interesting issue that has not been explored is whether the adverse effects of a dietary pattern might be associated with physical activity and fitness among older adults; hence, the aims of this study were to assess prevailing food patterns and its association with physical activity and fitness among Spanish older adults.

## 2. Methods

### 2.1. Study Design, Population and Ethics

The sample consisted of 380 participants (54% women) engaged in a cross-sectional study conducted from 2013 to 2014 into two different areas (Balearic Islands and Madrid) investigating the effect of the lifestyle factors on health of older adults living in Spain. Men aged between 55 and 80 and women aged between 60 and 80 were recruited in social and municipal clubs, health centers and sport clubs. Exclusion criteria included being institutionalized, suffering from a physical or mental illness which would have limited their participation in physical fitness or their ability to respond to questionnaires, chronic alcoholism or drug addiction and intake of drugs for clinical research over the past year. The study was conducted according to the guidelines of the Declaration of Helsinki, and all procedures were approved by the Ethics Committee of the Technical University of Madrid. Written informed consent was obtained from all participants.

### 2.2. Socio-Economic Determinants

A validated questionnaire [21] including the following questions was used: age, marital status, educational level, and income. Data were grouped in binary categories as follows: (a) marital status: uncoupled (single, unmarried, divorced or widowed), and coupled (i.e., including married and unmarried, divorced or widowed actually living with a partner); (b) educational level: primary and secondary, or college-level education; and (c) income: <900 €/month, and ≥900 €/month.

### 2.3. Body Composition

Anthropometric measurements were performed by well-trained observers to minimize the inter-observer coefficients of variation. Height was determined using a mobile anthropometer (Seca 213, SECA Deutchland, Hamburg, Germany) to the nearest millimeter, with the participant’s head maintained in the Frankfort Horizontal Plane position. This plane is defined as passing through the tragion (center of the ear hole) of each ear and the lowest point of the interior ridge of each eye. Body weight and body fat were determined using a Segmental Body Composition Analyzer (Tanita BC-418, Tanita, Tokyo, Japan). The participants were weighed in bare feet and light clothes, and subtracting 0.6 kg for their clothes. Waist circumference (WC) was measured as the smallest horizontal girth between the costal margins and the iliac crests at minimal respiration using a flexible, non-extensible plastic tape with 0.1 cm precision (Kawe 43972, Kirchner & Wilhelm GmbH + Co. KG, Asperg, Germany). Weight and height measures were used to calculate body mass index (BMI, kg/m^2^). Obesity was defined as BMI ≥ 30 kg/m^2^ [22]. Overfat was defined according to Gallagher et al. [23]. WC and height measures were used to calculate waist-to-height ratio (WHtR). Abdominal-obesity was defined as a WHtR ≥ 0.5 [24].

### 2.4. Dietary Patterns Derived from Food Consumption Data

Data on dietary intake were assessed using a validated semi-quantitative 137-item food frequency questionnaire (FFQ) [25]. For each item, a typical portion size was included, and consumption frequencies were registered in 9 categories that ranged from “never or almost never” to “6 times/day”. Daily food consumption was estimated by multiplying the portion size of each food item by its consumption frequency. Identification of under-reporters of food intake was made using two non-consecutive 24 h diet recalls conducted any two-days of the week from Monday to Sunday to account for day-to-day intake variability. Volumes and portion sizes were reported with the aid of a book of photographs [26]. Conversion of food into energy intake (EI) and nutrients was made using a computer program (ALIMENTA^®^; NUCOX, Palma, Spain) based on Spanish [27,28] and European [29] food composition tables, and complemented with food composition data available for Majorcan food items [30]. Licensed dietitians administered the recalls and verified and quantified the food records. Under-reporters (EI/basal metabolic rate <0.96) were not considered in the food dietary patterns analysis (*n* = 10) and the final sample comprised 370 participants [31].

The 137 food items included in the FFQ were grouped into 50 predefined food categories. A factor analysis using the principal components analysis as a method of extracting factors was applied to these 50 categories in order to identify a reduced number of factors that could explain the maximum proportion of the variance from the original groups. The factors were rotated by orthogonal transformation (Varimax rotation) to achieve a simpler structure with greater interpretability. We selected 2 factors that had eigenvalues exceeding 2.5 on the scree-plot and accounted for 14.0% (8.35% + 5.66%) of the total variance. Our results agree with a previous report conducted in men aged 55–80 years and women aged 60–80 years from the Mediterranean area, where the 2 factors selected accounted for 13% of the total variance [32]. Food groups with absolute loading ≥0.250 were considered relevant factors of the identified patterns (Table 1). The first factor (called “Western dietary pattern”, WDP) was positively associated with frequent intakes of whole dairy products, meats (i.e., red meat, viscera, low fat processed meat, and high fat processed meat), seafood, potatoes, refined grain bread, rice, pasta, non-extra virgin olive oils, non-olive oils, bakery (i.e., homemade and also commercial bakery), chocolate, sugar, processed meals, sauces, spices, salt, and commercial juices; and negatively associated with low fat dairy products, white fish, and olives and extra-virgin olive oil. The second factor (called “Mediterranean dietary pattern”, MDP) was positively associated with frequent intakes of white and blue-fish, canned fish/seafood, fruit, canned and dried fruit, jam, vegetables, potatoes, nuts, legumes, whole grain bread, rice, olives and extra-virgin olive oil, honey, and spices. For each pattern, each participant received a score coefficient. The factor score coefficients were estimated by the Anderson-Rubin method [33], which produces uncorrelated scores with a mean of zero and a standard deviation of 1. A higher score indicated a higher adherence to the respective dietary pattern.

### 2.5. Physical Activity

Physical activity (PA) data was estimated using the validated Spanish version of the Minnesota Leisure Time Physical Activity Questionnaire [34,35]. This questionnaire, taken by interview with trained research assistants, measures leisure time physical activities (LTPA) including household activities, over the previous 12 months. This questionnaire was used to estimate PA levels by using metabolic equivalents of task (MET) [36]. MET are calculated by multiplying the intensity (showed by the MET-score) and the duration spent on that activity (measured in minutes). The MET-score can be derived from tables (the Compendium of Physical Activities) [37] that show the intensity of the activity relative to resting. MET-minutes spent on PA refer to the energy that is spent on activities, over and above existing levels of resting energy expenditure. The resulting quantitative MET for each participant were categorized into tertiles (T1: low, T2: medium, T3: high).

## 3. Physical Fitness

Fitness measurements included grip muscular strength, lower and upper body strength, agility/dynamic balance, gait speed, and aerobic endurance [38,39].

Grip muscular strength was measured using a digital handheld dynamometer (T.K.K. 5401 Grip-D; Takey, Tokyo, Japan). Participants were instructed to stand up-right with the dynamometer beside, but not against their body. Measurements were performed two times for each hand. The best of all attempts was used for performance analysis.

Lower body strength was assessed by the 30-s Chair Stand Test. The 30-s Chair Stand Test consists of standing up and sitting down from a chair as many times as possible within 30 s. Initially, participants were seated on the chair with their back in an upright position. They were instructed to look straight forward and to rise after the “1, 2, 3, go” command at their own preferred speed with their arms folded across their chest.

Upper body strength was assessed by the 30-s Arm Curl Test. On a signal, participants were instructed to flex and stand the holding hand weight (men: 4 kg; women: 2.5 kg) through the complete range of motion, as many times as possible in 30 s. Measurements were performed two times for each arm. The best of all attempts was used for further analysis.

Agility/dynamic balance was assessed by the 8-foot Time Up-and-Go test. Participants were instructed to rise from a chair without the use of arms, walk around the cone placed 2.45 m from the chair, and return to the original sitting position. Further instructions were to complete the test as quickly as possible and taking care not to run. Measurement was performed two times and the best attempt was used for analysis.

Gait speed was performed over 30 m at the participants’ usual place. Participants were instructed to complete the test as quickly as possible, while taking care not to run. Measurement was performed two times and the best attempt was used for analysis.

Aerobic endurance was performed as the average of speed during a 6-min Walking test. Participants were instructed to walk at their usual pace around the cones placed 4.6 m apart.

### Statistics

Analyses were performed with the SPSS statistical software package version 24.0 (SPSS Inc., Chicago, IL, USA). The resulting quantitative scores of both selected food group (dietary patterns) for each participant were categorized into quartiles. Characteristics and physical fitness of participants according to their quartiles of adherence to both dietary patterns were analyzed (Table 2, Table 3, Table 4 and Table 5). Physical fitness results differed between genders (data not shown); accordingly, all these analyses were stratified by sex. The difference in prevalence across WDP and MDP quartiles was examined by using χ^2^ (Table 2, Table 3 and Table 4). Logistic regression analysis was also used to examine the possible association between socioeconomic and anthropometric characteristics of participants (independent variables) and adherence to both selected dietary patterns, WDP and MDP (dependent variables) after adjustment for age (Table 2 and Table 3). We modeled the probability to obtain a higher score of WDP and MDP (fourth quartile vs. the others) reflecting a higher adherence to the respective dietary pattern. Logistic regression models were also used to examine the possible association between tertiles of LTPA (independent variables) and adherence to both selected dietary patterns (dependent variables) after adjustment for age, body fat and WHtR (Table 4). Finally, differences in physical fitness score means (95% of confidence interval) according to both selected dietary patterns were compared using an analysis of covariance (ANCOVA) after adjustment for potential confounding factors (Model 1: age; Model 2: age, body fat, and WHtR; Model 3: age, body fat, WHtR, and METs) (Table 5). In Model 1, the differences were established by the Bonferroni’s post-hoc test for multiple comparisons. A *p* value ≤ 0.05 was considered statistically significant.

## 4. Results

The characteristics of participants by quartiles of the WDP and the MDP stratified by gender are shown in Table 2 and Table 3, respectively. Participants with a higher adherence to the WDP (fourth quartile vs. the others) were more likely to be men. Men with a higher adherence to the WDP were more likely to have a primary or secondary-level education. Moreover, women with a higher adherence to the WDP were more likely to have an income <900 €/month. Participants with a higher adherence to the MDP were also less likely to have abdominal obesity.

No significant association was found between the WDP and the LTPA practice (Table 4). However, participants in the MDP’s fourth quartile were more likely to be classified in the second (men) and third (men and women) tertile of LTPA, and these associations remained significant after adjustments by age, body fat and WHtR.

After age adjustment, a negative significant association was found in both sexes between the 30-s Chair stand and 6-min walking tests with the WDP (Table 5). Contrarily, a positive significant association was found between the 30-m Gait speed and the 8-foot Time Up-and-Go (except in men) with the WDP (*P* < 0.001). However, while the 30-s Chair stand (in men) and the 6-min walking tests (men: *P* = 0.082, women: *P* = 0.022) were positively associated with the MDP, the 8-foot Time Up-and-Go (in men) and the 30-m gait speed (men: *P* = 0.005, women: *P* = 0.088) tests were negatively associated with the MDP. After additional adjustments for body fat and WHtR, the associations with the WDP remained significant, and only the association between the 30-m gait speed test and the MDP in men remained significant.

## 5. Discussion

The main finding of the present study is that participants classified in the fourth quartile of a MDP (i.e., characterized by high intake of whole grain bread, rice, fish, fruits, vegetables, potatoes, legumes, nuts, olives and extra-virgin olive oil, honey, jam, and spices) were more likely to practice LTPA, and this association was independent of body composition. Additionally, MDP was associated with a faster gait speed in men. In contrast, a WDP (i.e., characterized by high intake of refined grain bread, rice, pasta, whole dairy products, seafood, meats, potatoes, non-extra virgin olive oils and non-olive oil fats, processed meals, bakery, chocolate, sugar, sauces, spices, salt, and commercial juices) was associated with slower gait speed, and lower body strength, agility (in women), and aerobic endurance.

A healthy diet, such as a MDP, has usually been associated with a healthier lifestyle [40], while a WDP has been linked to less healthy behaviors [41]. Healthy diets have also been inversely associated with frailty [1,5]. Shikany et al. [9] found that among 5925 men aged ≥65 years enrolled in the Osteoporotic Fractures in Men Study (MrOS), overall diet quality was inversely associated with current and future frailty status. Several cross-sectional [1,8] and longitudinal [5,6] studies have also observed an association of higher adherence to the MD—measured with the Mediterranean Diet Score (MDS) and/or the Mediterranean Diet Adherence Screener (MEDAS) score—with lower odds of developing frailty compared with those with lower adherence. These studies associated higher adherence to the MD with lower risk of low physical activity [5,8] and low walking speed [5,6,8], but no with feelings of exhaustion and poor muscle strength [5,8]. Faster walking speed in community-dwelling elderly people (aged ≥70 years) with higher adherence to the MD—assessed by the MDS—after adjustment for potential confounders (i.e., age, race, sex, site, education, smoking, physical activity, energy intake, health status, depression and cognitive score) has also been reported [4]. These differences remained significant over eight years, suggesting a long-term effect of diet on mobility performance with aging [4]. Similarly, an inverse association between a prudent dietary pattern (i.e., characterized by high intake of olive oil, vegetables, potatoes, legumes, blue fish, pasta, and meat) comparable to the MDP, and frailty has been reported [41]. In the present study, while a higher adherence to the MDP was associated with faster gait speed in men, the WDP was associated with a slower gait speed in both genders after adjusting for potential confounders such as age, body fat, WHtR and METs.

Lately, an inverse association between the risk of frailty with consumption of fish and fruit has been pointed out [6]. It was also found that community-dwelling older adults (aged 59 to 73) whose diets were characterized by high consumption of fruit, vegetables, whole wheat bread and fatty fish had higher handgrip strength [42]. Furthermore, it was also reported that while consumption of lean fish and shellfish was not related to grip strength, in comparison with no consumption, consuming fatty fish more than once per week was associated with a gain in handgrip strength of around 3 kg [42]. In our study, a WDP, characterized by seafood but no fish consumption, was associated with lower body strength, agility (in women) and aerobic endurance. In this line, Rousseau et al. found modest positive significant correlation between reported omega-3 fatty acids (i.e., the main source of the parent compounds responsible for omega-3 fatty acids metabolites) and leg strength, but modest negative significant correlation between reported omega-3 fatty acids and time to rise from a chair [43].

The association of dairy consumption (milk, yogurt and cheese) with physical fitness in 1456 older women (aged 70 to 85 years) showed that those in the third tertile had significantly greater appendicular skeletal muscle mass, greater handgrip strength and 26% lower odds for a poor Timed Up-and-Go test, suggesting an association of high dairy intake with better physical fitness in this population [44]. In our study, while the MDP was not associated with dairy consumption, the WDP was characterized by high intake of whole dairy products. Nevertheless, WDP was positively associated with 8-foot time up-and-go test in women, suggesting poor agility among women with higher adherence to the WDP.

Therefore, our results seem to indicate that the MDP might maintain a protective effect on healthy physical fitness, but that WDP might be a contributor to less physical fitness, and therefore, to frailty. WDP evinced an increased risk of slow gait speed and lower body strength, agility and aerobic endurance, as has been previously observed [41].

### Strengths and Limitations

This study has several strengths. Objective and validated physical fitness measurements were obtained in healthy Spanish older adults. The associations of healthy and unhealthy dietary patterns (MDP and WDP, respectively) and physical fitness were also assessed in this population, controlling potential confounding factors. This study has also several limitations. First, the present cross-sectional design gives limited ability to elucidate causal relationship between decline in physical performance and dietary patterns. Moreover, because of the cross-sectional study design we cannot say anything about if our WDP contributes to the occurrence or diagnosis of frailty. Second, the potential lack of generalizability of results due to the select nature on including participants from only two places as well as the sample size obtained. Third, despite the guidance of licensed dietitians, diet was self-reported. Although a validated FFQ was used, we cannot rule out some recall bias.

## 6. Conclusions

The MDP is associated with more time spent on LTPA, and this association was independent of body composition and a fast gait speed in men. The WDP is associated with slower gait speed and lower body strength, agility and aerobic endurance. Therefore, adherence to MDP contributes to a protective effect on healthy physical fitness, and WDP may be a frailty contributor. Older adults are priority targets for action. Then, they should recover the MDP and its health benefits. Early management of healthy nutrition patterns, as the MDP is, and a multicomponent training exercise program (strength, flexibility, agility, and endurance) should be encouraged to maintain physical fitness and health among older adults.

## Figures and Tables

**Table 1:** Factor-loading matrix for the two major dietary patterns among older adults.

	Western Dietary Pattern	Mediterranean Dietary Pattern
Percent of total variance	8.35	5.66
Whole dairy products	**0.495**	-
Low fat dairy products	−0.324	-
Eggs	-	-
White meat	-	-
Red meat	**0.516**	-
Viscera	0.403	-
Low fat processed meat	0.335	-
High fat processed meat	**0.562**	-
White fish	−0.354	0.293
Bluefish	-	**0.516**
Salted fish	-	-
Seafood	0.316	-
Canned fish/seafood	-	-
Fruits	-	0.369
Canned fruit	-	-
Dried fruit	-	0.290
Jam	-	0.281
Vegetables	-	**0.674**
Potatoes	**0.497**	0.377
Nuts	-	**0.430**
Legumes	-	**0.523**
Whole grain bread	-	0.271
Refined grain bread	0.358	-
Refined cereals	-	-
Whole cereals	-	-
Rice	0.403	0.320
Pasta	0.400	-
Olives and extra virgin olive oil	−0.290	0.284
Other olive oils	**0.419**	-
Other oils	0.252	-
Other fats	-	-
Homemade bakery	0.333	-
Commercial bakery	**0.473**	-
Chocolate	0.297	-
Sugar	**0.496**	-
Honey	-	0.345
Processed meals	**0.417**	-
Snacks	-	-
Sauces	0.349	-
Spices	0.266	0.328
Salt	0.391	-
Soda	-	-
Diet soda	-	-
Isotonic beverages	-	-
Commercial juices	0.273	-
Coffee and tea	-	-
Red wine	-	-
Other wines and cava	-	-
Beer	-	-
Distilled beverage	-	-

Values of <0.250 were excluded from the table for simplicity; those with loadings ≥0.400 are in bold.

**Table 2:** Characteristics of participants according to quartiles of adherence to Western Dietary Pattern (WDP) ^1,2^.

	Western Dietary Pattern (Quartiles)					Q4 vs. Q1 + 2 + 3 (ref.)
	Q1	Q2	Q3	Q4	*P*	OR (95% CI)
***Sex***	***n* = 92**	***n* = 93**	***n* = 93**	***n* = 92**		
Men	28 (30.4)	35 (37.6)	39 (41.9)	65 (70.7)	<0.001	1.00 (ref.)
Women	64 (69.6)	58 (62.4)	54 (58.1)	27 (29.3)		0.27 (0.16–0.45) ***
***Men***	***n* = 28**	***n* = 35**	***n* = 39**	***n* = 65**		
Marital status						
Coupled	25 (89.3)	30 (85.7)	32 (82.1)	58 (89.2)	0.731	1.00 (ref.)
Uncoupled	3 (10.7)	5 (14.3)	7 (17.9)	7 (10.8)		0.69 (0.27–1.82)
Educational level						
Primary or secondary	17 (60.7)	18 (51.4)	28 (71.8)	50 (76.9)	0.053	1.00 (ref.)
College	11 (39.3)	17 (48.6)	11 (28.2)	15 (23.1)		0.44 (0.22–0.90) *
Income						
<900 €/month	3 (10.7)	4 (11.4)	6 (15.4)	12 (18.5)	0.712	1.00 (ref.)
≥900 €/month	25 (89.3)	31 (88.6)	33 (84.6)	53 (81.5)		0.55 (0.23–1.32)
Obesity						
Non-obese	25 (89.3)	25 (71.4)	32 (82.1)	53 (81.5)	0.341	1.00 (ref.)
Obese	3 (10.7)	10 (28.6)	7 (17.9)	12 (18.5)		0.94 (0.42–2.09)
Body fat						
Non-overfat	11 (39.3)	14 (40.0)	12 (30.8)	20 (30.8)	0.708	1.00 (ref.)
Overfat	17 (60.7)	21 (60.0)	27 (69.2)	45 (69.2)		1.24 (0.63–2.41)
Abdominal obesity						
Non-abdominally obese	3 (10.7)	5 (14.3)	4 (10.3)	11 (16.9)	0.758	1.00 (ref.)
Abdominally obese	25 (89.3)	30 (85.7)	35 (89.7)	54 (83.1)		0.76 (0.30–1.87)
***Women***	***n* = 64**	***n* = 58**	***n* = 54**	***n* = 27**		
Marital status						
Coupled	32 (50.0)	39 (67.2)	36 (66.7)	22 (81.5)	0.025	1.00 (ref.)
Uncoupled	32 (50.0)	19 (32.8)	18 (33.3)	5 (18.5)		0.38 (0.13–1.08)
Educational level						
Primary or secondary	48 (75.0)	41 (70.7)	50 (92.6)	23 (85.2)	0.020	1.00 (ref.)
University	16 (25.0)	17 (29.3)	4 (7.4)	4 (14.8)		0.63 (0.21–1.95)
Income						
<900 €/month	31 (48.4)	25 (43.1)	29 (53.7)	20 (74.1)	0.058	1.00 (ref.)
≥900 €/month	33 (51.6)	33 (56.9)	25 (46.3)	7 (25.9)		0.30 (0.12–0.76) *
Obesity						
Non-obese	52 (81.3)	43 (74.1)	44 (81.5)	21 (77.8)	0.745	1.00 (ref.)
Obese	12 (18.8)	15 (25.9)	10 (18.5)	6 (22.2)		1.01 (0.38–2.69)
Body fat						
Non-overfat	24 (37.5)	23 (39.7)	22 (40.7)	14 (51.9)	0.641	1.00 (ref.)
Overfat	40 (62.5)	35 (60.3)	32 (59.3)	13 (48.1)		0.60 (0.27–1.37)
Abdominal obesity						
Non-abdominally obese	23 (35.9)	16 (27.6)	11 (20.4)	12 (44.4)	0.102	1.00 (ref.)
Abdominally obese	41 (64.1)	42 (72.4)	43 (79.6)	15 (55.6)		0.50 (0.22–1.15)

Abbreviations: OR, odds ratio; CI, confidence interval; BMI, body mass index; WHtR, waist-to-height ratio; ref., reference. ^1^ Significant differences in prevalence were calculated by means of χ^2^; ^2^ Binary logistic regression analysis considering the effect of one explanatory variable after adjustment for age. Q4 vs. Q1 + 2 + 3 (ref.): * *p* < 0.05; *** *p* < 0.001.

**Table 3:** Characteristics of participants according to quartiles of adherence to Mediterranean Dietary Pattern (MDP) ^1,2^.

	Mediterranean Dietary Pattern (Quartiles)					Q4 vs. Q1 + 2 + 3 (ref.)
	Q1	Q2	Q3	Q4	*P*	OR (95% CI)
***Sex***	***n* = 92**	***n* = 93**	***n* = 93**	***n* = 92**		
Men						1.00 (ref.)
Women	46 (50.0)	54 (58.1)	54 (58.1)	49 (53.3)	0.627	0.82 (0.50–1.33)
***Men***	***n* = 46**	***n* = 39**	***n* = 39**	***n* = 43**		
Marital status						
Coupled	39 (84.8)	35 (89.7)	35 (89.7)	36 (83.7)	0.774	1.00 (ref.)
Uncoupled	7 (15.2)	4 (10.3)	4 (10.3)	7 (16.3)		1.42 (0.54–3.78)
Educational level						
Primary or secondary	31 (67.4)	27 (69.2)	28 (71.8)	27 (62.8)	0.846	1.00 (ref.)
College	15 (32.6)	12 (30.8)	11 (28.2)	16 (37.2)		1.44 (0.69–3.01)
Income						
<900 €/month	10 (21.7)	5 (12.8)	4 (10.3)	6 (14.0)	0.473	1.00 (ref.)
≥900 €/month	36 (78.3)	34 (87.2)	35 (89.7)	37 (86.0)		1.25 (0.46–3.44)
Obesity						
Non-obese	37 (80.4)	30 (76.9)	34 (87.2)	34 (79.1)	0.685	1.00 (ref.)
Obese	9 (19.6)	9 (23.1)	5 (12.8)	9 (20.9)		1.15 (0.49–2.75)
Body fat						
Non-overfat	12 (26.1)	12 (30.8)	12 (30.8)	21 (48.4)	0.119	1.00 (ref.)
Overfat	34 (73.9)	27 (69.2)	27 (69.2)	22 (51.2)		0.44 (0.21–0.90) *
Abdominal obesity						
Non-abdominally obese	3 (6.5)	3 (7.7)	5 (12.8)	12 (27.9)	0.015	1.00 (ref.)
Abdominally obese	43 (93.5)	36 (92.3)	34 (87.2)	31 (72.1)		0.26 (0.10–0.73) *
***Women***	***n* = 46**	***n* = 54**	***n* = 54**	***n* = 49**		
Marital status						
Coupled	32 (69.6)	35 (64.8)	30 (55.6)	32 (65.3)	0.510	1.00 (ref.)
Uncoupled	14 (30.4)	19 (35.2)	24 (44.4)	17 (34.7)		0.73 (0.36–1.48)
Educational level						
Primary or secondary	38 (82.6)	42 (77.8)	45 (83.3)	37 (75.5)	0.720	1.00 (ref.)
University	8 (17.4)	12 (22.2)	9 (16.7)	12 (24.5)		1.50 (0.69-3.27)
Income						
<900 €/month	26 (56.5)	26 (48.1)	28 (51.9)	25 (51.0)	0.871	1.00 (ref.)
≥900 €/month	20 (43.5)	28 (51.9)	26 (48.1)	24 (49.0)		1.12 (0.58–2.16)
Obesity						
Non-obese	29 (63.0)	44 (81.5)	46 (85.2)	41 (83.7)	0.028	1.00 (ref.)
Obese	17 (37.0)	10 (18.5)	8 (14.8)	8 (16.3)		0.72 (0.31–1.70)
Body fat						
Non-overfat	10 (21.7)	21 (38.9)	30 (55.6)	22 (44.9)	0.007	1.00 (ref.)
Overfat	36 (78.3)	33 (61.1)	24 (44.4)	27 (55.1)		0.79 (0.41–1.52)
Abdominal obesity						
Non-abdominally obese	10 (21.7)	12 (22.2)	18 (33.3)	22 (44.9)	0.038	1.00 (ref.)
Abdominally obese	36 (78.3)	42 (77.8)	36 (66.7)	27 (55.1)		0.43 (0.23–0.80) **

Abbreviations: OR, odds ratio; CI, confidence interval; BMI, body mass index; WHtR, waist-to-height ratio; ref., reference. ^1^ Significant differences in prevalence were calculated by means of χ^2^; ^2^ Binary logistic regression analysis considering the effect of one explanatory variable after adjustment for age. Q4 vs. Q1 + 2 + 3 (ref.): * *p* < 0.05; ** *p* < 0.01.

**Table 4:** Association between leisure-time physical activity practice (LTPA) and adherence to Western Dietary Pattern (WDP) and Mediterranean Dietary Pattern (MDP) among participants ^1,2^.

		WDP				Q4 vs. Q1 + 2 + 3 (ref.)
LTPA	*n*	Q1	Q2	Q3	Q4	OR (95% CI)
Men		*n* = 28	*n* = 35	*n* = 39	*n* = 65	
T1: Low	55	11 (20.0)	15 (27.3)	9 (16.4)	20 (36.4)	1.00 (ref.)
T2: Medium	57	6 (10.5)	11 (19.3)	17 (29.8)	23 (40.4)	1.25 (0.58–2.72)
T3: High	55	11 (20.0)	9 (16.4)	13 (23.6)	22 (40.0)	1.17 (0.52–2.63)
*P*		0.423				0.848
**Women**		***n* = 64**	***n* = 58**	***n* = 54**	***n* = 27**	
T1: Low	67	29 (43.3)	19 (28.4)	13 (19.4)	6 (9.0)	1.00 (ref.)
T2: Medium	68	16 (23.5)	21 (30.9)	22 (32.4)	9 (13.2)	1.50 (0.50–4.55)
T3: High	68	19 (27.9)	18 (26.5)	19 (27.9)	12 (17.6)	2.27 (0.78–6.63)
*P*		0.177				0.316
**LTPA**		**MDP**				**Q4 vs. Q1 + 2 + 3 (ref.)**
	***n***	**Q1**	**Q2**	**Q3**	**Q4**	**OR (95% CI)**
**Men**		***n* = 46**	***n* = 39**	***n* = 39**	***n* = 43**	
T1: Low	55	19 (34.5)	17 (30.9)	13 (23.6)	6 (10.9)	1.00 (ref.)
T2: Medium	57	10 (17.5)	13 (22.8)	14 (24.6)	20 (35.1)	4.13 (1.48–11.51) **
T3: High	55	17 (30.9)	9 (16.4)	12 (21.8)	17 (30.9)	3.03 (1.05–8.74) *
*P*		0.042				0.024
**Women**		***n* = 46**	***n* = 54**	***n* = 54**	***n* = 49**	
T1: Low	67	40 (32.8)	39 (32.0)	27 (22.1)	16 (13.1)	1.00 (ref.)
T2: Medium	68	25 (20.0)	30 (24.0)	34 (27.2)	36 (28.8)	1.71 (0.69–4.23)
T3: High	68	27 (22.0)	24 (19.5)	32 (26.0)	40 (32.5)	3.06 (1.26–7.42) *
*P*		0.063				0.042

Abbreviations: T, tertile; OR, odds ratio; CI, confidence interval; ref., reference. Values are expressed as *n* (%) and OR (95% CI). ^1^ Significant differences in prevalence were calculated by means of χ^2^; ^2^ Logistic regression analysis comparing LTPA between the fourth quartile of adherence to both dietary pattern (WDP4 and MDP4) and the others quartiles as reference value (WDP1 + WDP2 + WDP3 and MDP1 + MDP2 + MDP3) after adjustment for age, body fat, and WHtR (continuous variables). Q4 vs. Q1 + 2 + 3 (ref.): * *p* < 0.05; ** *p* < 0.01.

**Table 5:** Mean (95% CI) of physical fitness scores according to quartiles of adherence to Western Dietary Pattern (WDP) and Mediterranean Dietary Pattern (MDP) among participants ^1,2,3^.

	**WDP1**	**WDP2**	**WDP3**	**WDP4**	***P*^1^**	***P*^2^**	***P*^3^**
**Men**	***n* = 28**	***n* = 35**	***n* = 39**	***n* = 65**			
Handgrip strength (kg)	38.7 (36.4–41.0)	39.6 (37.5–41.6)	36.9 (35.0–38.9)	37.9 (36.4–39.4)	0.294	0.211	0.215
30-s Chair stand (No. of stands)	17.1 (15.8–18.4) ^a^	16.0 (14.8–17.2)	15.6 (14.5–16.7)	14.9 (14.0–15.7) ^a^	0.042	0.022	0.022
Arm Curl (No. of reps)	18.8 (17.1–20.5)	19.0 (17.4–20.5)	19.7 (18.2–21.1)	18.9 (17.8–20.1)	0.828	0.899	0.892
8-foot Time Up-and-Go (s)	4.7 (4.4–5.0)	4.5 (4.2–4.8)	4.7 (4.4–4.9)	4.9 (4.7–5.0)	0.174	0.139	0.128
30-m Gait speed (s)	12.6 (11.7–13.6) ^a^	12.6 (11.7–13.4)	13.0 (12.2–13.8)	14.5 (13.8–15.1) ^a^	0.001	<0.001	<0.001
6-min Walking test (m)	693.9 (658.3–729.4) ^a^	675.1 (643.4–706.8) ^b^	652.8 (622.8–682.8)	617.2 (593.8–640.6) ^a,b^	0.002	<0.001	<0.001
**Women**	***n* = 64**	***n* = 58**	***n* = 54**	***n* = 27**			
Handgrip strength (kg)	22.5 (21.5–23.5)	21.8 (20.7–22.9)	21.1 (20.0–22.2)	20.7 (19.1–22.3)	0.165	0.104	0.109
30-s Chair stand (No. of stands)	15.2 (14.4–15.9) ^a^	14.0 (13.2–14.8)	13.9 (13.1–14.8)	12.1 (11.0–13.3) ^a^	0.001	<0.001	<0.001
Arm Curl (No. of reps)	16.4 (15.6–17.3)	16.4 (15.5–17.3)	16.1 (15.2–17.1)	15.9 (14.6–17.2)	0.901	0.875	0.796
8-foot Time Up-and-Go (s)	5.0 (4.8–5.2) ^a,b^	5.3 (5.1–5.5)	5.5 (5.3–5.7) ^d^	5.8 (5.4–6.1) ^a,d^	<0.001	<0.001	<0.001
30-m Gait speed (s)	15.1 (14.5–15.7) ^a,b^	16.3 (15.7–16.9)	17.0 (16.3–17.6) ^d^	17.2 (16.3–18.1) ^a,d^	<0.001	<0.001	<0.001
6-min Walking test (m)	600.9 (583.9–617.9) ^a,b,c^	551.6 (533.8–569.4) ^c^	533.6 (515.1–552.1) ^d^	528.1 (501.8–554.3) ^a,d^	<0.001	<0.001	<0.001
	**MDP1**	**MDP2**	**MDP3**	**MDP4**	***P*^1^**	***P*^2^**	***P*^3^**
**Men**	***n* = 46**	***n* = 39**	***n* = 39**	***n* = 43**			
Handgrip strength (kg)	38.5 (36.7–40.3)	37.9 (35.9–39.8)	37.3 (35.4–39.3)	38.8 (36.9–40.6)	0.710	0.808	0.802
30-s Chair stand (No. of stands)	14.9 (13.9–16.0) ^a^	15.2 (14.1–16.3)	15.4 (14.3–16.5)	17.1 (16.0–18.1) ^a^	0.023	0.120	0.135
Arm Curl (No. of reps)	18.6 (17.3–19.9)	19.3 (17.9–20.8)	18.3 (16.9–19.8)	20.1 (18.7–21.5)	0.278	0.491	0.501
8-foot Time Up-and-Go (s)	4.8 (4.5–5.0)	4.8 (4.6–5.1)	4.9 (4.6–5.1) ^d^	4.4 (4.2–4.6) ^d^	0.024	0.120	0.131
30-m Gait speed (s)	13.5 (12.7–14.2)	13.7 (12.9–14.5)	14.3 (13.5–15.1) ^d^	12.3 (11.5–13.0) ^d^	0.005	0.024	0.026
6-min Walking test (m)	637.6 (609.2–665.9)	642.4 (611.6–673.2)	637.1 (606.2–667.9)	684.0 (654.5–713.4)	0.082	0.318	0.348
**Women**	***n* = 46**	***n* = 54**	***n* = 54**	***n* = 49**			
Handgrip strength (kg)	22.6 (21.4–23.9)	21.1 (20.0–22.3)	21.6 (20.5–22.8)	21.4 (20.2–22.6)	0.328	0.514	0.532
30-s Chair stand (No. of stands)	14.4 (13.5–15.3)	14.0 (13.1–14.9)	13.9 (13.0–14.7)	14.2 (13.3–15.1)	0.856	0.595	0.565
Arm Curl (No. of reps)	16.8 (15.8–17.9)	16.2 (15.3–17.2)	15.9 (14.9–16.8)	16.2 (15.3–17.2)	0.572	0.461	0.263
8-foot Time Up-and-Go (s)	5.4 (5.1–5.6)	5.4 (5.1–5.6)	5.5 (5.2–5.7)	5.1 (4.8–5.3)	0.214	0.378	0.425
30-m Gait speed (s)	16.6 (15.9–17.3)	16.2 (15.5–16.8)	16.6 (15.9–17.3)	15.5 (14.7–16.2)	0.088	0.206	0.284
6-min Walking test (m)	537.7 (516.3–559.1) ^a^	558.6 (538.9–578.3)	555.0 (535.4–574.7)	584.7 (563.9–605.5) ^a^	0.022	0.174	0.276

Statistical significant differences were assessed using analysis of covariance (ANCOVA) after adjustment for potential confounding factors (Model 1: age; Model 2: age, body fat, and WHtR; Model 3: age, body fat, WHtR, and METs (continuous variables)). Significant differences between pairs of means by Bonferroni’s post-hoc test after adjustment for age (*P* < 0.05): ^a^ DP1 vs. DP4; ^b^ DP2 vs. DP4; ^c^ DP1 vs. DP2; ^d^ DP3 vs. DP4.
